# A qualitative analysis of smokers’ perceptions about lung cancer screening

**DOI:** 10.1186/s12889-017-4496-0

**Published:** 2017-06-21

**Authors:** Lindsay Gressard, Amy S. DeGroff, Thomas B. Richards, Stephanie Melillo, Julia Kish-Doto, Christina L. Heminger, Elizabeth A. Rohan, Kristine Gabuten Allen

**Affiliations:** 1 Division of Cancer Prevention and Control, National Center for Chronic Disease Prevention and Health Promotion, Centers for Disease Control and Prevention, 4770 Buford Hwy NE, MS F-76, Atlanta, GA 30341-3717 USA; 2 RTI International, Research Triangle Park, NC USA; 3 The George Washington University, Washington, DC USA

**Keywords:** Lung cancer screening, Smokers, Barriers, Patient-provider communication

## Abstract

**Background:**

In 2013, the US Preventive Services Task Force (USPSTF) began recommending lung cancer screening for high risk smokers aged 55–80 years using low-dose computed tomography (CT) scan. In light of these updated recommendations, there is a need to understand smokers’ knowledge of and experiences with lung cancer screening in order to inform the design of patient education and tobacco cessation programs. The purpose of this study is to describe results of a qualitative study examining smokers’ perceptions around lung cancer screening tests.

**Methods:**

In 2009, prior to the release of the updated USPSTF recommendations, we conducted 12 120-min, gender-specific focus groups with 105 current smokers in Charlotte, North Carolina and Cincinnati, Ohio. Focus group facilitators asked participants about their experience with three lung cancer screening tests, including CT scan, chest x-ray, and sputum cytology. Focus group transcripts were transcribed and qualitatively analyzed using constant comparative methods.

**Results:**

Participants were 41–67 years-old, with a mean smoking history of 38.9 pack-years. Overall, 34.3% would meet the USPSTF’s current eligibility criteria for screening. Most participants were unaware of all three lung cancer screening tests. The few participants who had been screened recalled limited information about the test. Nevertheless, many participants expressed a strong desire to pursue lung cancer screening. Using the social ecological model for health promotion, we identified potential barriers to lung cancer screening at the 1) health care system level (cost of procedure, confusion around results), 2) cultural level (fatalistic beliefs, distrust of medical system), and 3) individual level (lack of knowledge, denial of risk, concerns about the procedure). Although this study was conducted prior to the updated USPSTF recommendations, these findings provide a baseline for future studies examining smokers’ perceptions of lung cancer screening.

**Conclusion:**

We recommend clear and patient-friendly educational tools to improve patient understanding of screening risks and benefits and the use of best practices to help smokers quit. Further qualitative studies are needed to assess changes in smokers’ perceptions as lung cancer screening with CT scan becomes more widely used in community practice.

**Electronic supplementary material:**

The online version of this article (doi:10.1186/s12889-017-4496-0) contains supplementary material, which is available to authorized users.

## Background

Recommendations for lung cancer screening have been rapidly evolving in the United States [1]. In 2011, the National Lung Screening Trial (NLST) reported a 20% decrease in mortality when low-dose computed tomography (CT) was used to screen people who had smoked at least one pack of cigarettes a day for 30 years and who were either current smokers or had quit within the past 15 years [2]. In 2013, the US Preventive Services Task Force (USPSTF) recommended annual screening with low-dose CT for adults aged 55–80 years who meet the NLST smoking history criteria noted above [3]. In 2015, the Centers for Medicare & Medicaid Services (CMS) approved coverage for the procedure [4].

Prior to these developments, however, a national survey in 2006–2007 found that some primary care physicians were ordering lung cancer screening tests before any professional organizations began recommending it [5]. The results of the survey raised concerns about inappropriate lung cancer screening. Among surveyed physicians, 55% had ordered chest x-rays, 22% had ordered low-dose CT scans, about 5% had ordered sputum cytology, and 38% did not order lung cancer screening [5]. Both chest x-rays and sputum cytology still lack sufficient evidence for recommendation by the USPSTF.

To better understand the use of lung cancer screening, we conducted a two-part study in 2009. In the first part of the study, we conducted five telephone focus groups with primary care physicians to identify factors influencing physicians’ decisions around lung cancer screening. [6]. Influential factors included perceptions of test effectiveness, attitudes toward screening guidelines, practice experiences, perceptions of patient’s risk, litigation concerns, insurance reimbursement, and patient request. In the second part of our study, we conducted in-person focus groups with cigarette smokers to assess their experience with lung cancer screening. The aim of this paper is to present a qualitative analysis of the focus groups with smokers in order to better understand smokers’ knowledge and perceptions around the three most commonly used lung cancer screening tests: CT scan, chest x-ray, and sputum cytology. This paper also presents a number of potential barriers to obtaining lung cancer screening which were uncovered during analysis.

Although the context for lung cancer screening has changed since our study was conducted, these results provide a baseline for future qualitative studies of smokers’ perceptions about lung cancer screening. The findings around smokers’ barriers to screening are particularly important given the challenges to lung cancer screening implementation that researchers and practitioners continue to identify, including patient access and disparities [7]. As lung cancer screening with low-dose CT is implemented alongside tobacco cessation initiatives, information about smokers’ perceptions of screening will continue to be critical in guiding the design and implementation of effective screening programs.

## Methods

For this study, we conducted 12 approximately 120-min, in-person focus groups with cigarette smokers—six in Charlotte, North Carolina, and six in Cincinnati, Ohio. In accordance with focus group implementation recommendations [8], each group had 8–9 participants, was limited to either men or women, and was led by a professional focus group moderator of the same sex who was employed by a marketing research firm. The study was supported and led by the Centers for Disease Control and Prevention (CDC) in collaboration with Research Triangle Institute International (RTI). The institutional review boards of both organizations reviewed and approved the protocol for the study. The Office of Management and Budget approved data collection (ICR 200811–0920-003). Participants provided written consent and received $75 for their participation.

### Participants

Two companies specializing in conducting focus groups, AOC Marketing Research in Charlotte and Fields Research in Cincinnati, used existing databases to recruit 105 smokers (51 women and 54 men) for participation in this study. All participants met the following inclusion criteria: aged 40–70 years, current smoker with a history of smoking at least one pack of cigarettes per day for 20 years, no previous history of cancer or major lung conditions, English speaker, currently insured, and had a physical exam within the past 2 years.

### Data collection

Moderators used a semi-structured guide (included in Additional file 1) to facilitate discussion on participants’ perceptions about and use of lung cancer screening tests. After initial discussion about preventive health care and health screenings in general, participants were asked: “Did your doctor ever talk to you about screening tests for your lungs—that is, screening tests for lung cancer?” Next, participants were shown pictures of equipment for CT scans, chest x-rays, and sputum cytology tests. For each test, participants were asked a series of questions to determine whether they knew the test could be used for lung cancer screening, what their doctor had told them about the test, and whether their doctor had recommended the test for them. In order to determine if a test was used for screening, the facilitator asked the following question: "Was the test done as part of your routine care or check-up?" To identify diagnostic tests, the facilitator asked: "Did you have that test because of a particular problem or symptom?" When asking questions about CT scans, the facilitators did not name specific types of CT scans, such as a low-dose CT scans, to prevent confusion.

At the end of each focus group, the facilitator told participants that the USPSTF currently did not recommend screening for lung cancer with any test at that time, but that studies were underway to continue to assess the effectiveness of CT scans. All participants were then given information about their state’s smoking quitline, as well as websites for CDC and the National Cancer Institute.

### Data analysis

All focus groups were audio recorded. Our research team transcribed each focus group, vetted the transcript against the original audio recording, and entered the data into a qualitative analysis program, ATLAS.ti (version 5.6.1). Following the grounded theory approach, we used the constant comparative method of qualitative analysis to create and define inductive codes emerging from the data [9]. We refined the final set of codes after examining transcripts for new themes and expanding on existing themes. We developed a detailed codebook that followed a standard structure [10]. Two researchers jointly coded five transcripts using the codebook, reaching 83% agreement in assigned codes. Once all transcripts were coded, a team of three researchers created data matrices to identify and analyze thematic patterns. Upon completion of analysis, we organized data pertaining to barriers to screening by using a modified version of the social ecological model for health promotion to categorize barriers at the health care system, cultural, and individual levels [11].

## Results

### Demographics

The 105 focus group participants were divided relatively evenly by sex and study location (Table 1). They ranged in age from 41 to 67 years old, with more than 60% being younger than age 55 years. Although the majority were white, 39.0% were African American. Nearly two-thirds of participants had attended some college or more. All participants were current smokers who had smoked at least 20 pack-years, with one pack-year being the equivalent of smoking one pack every day for a year. More than 75% reported smoking at least 30 pack-years. Overall, 34.3% of participants would meet the current USPSTF and CMS eligibility criteria for referral for lung cancer screening with low-dose CT (i.e., at least 55 years of age and at least 30 pack-years).

**Table 1:** Focus group participants’ demographic characteristics, 2009

	Women		Men		Total
Focus group location	Charlotte, North Carolina	Cincinnati, Ohio	Charlotte, North Carolina	Cincinnati, Ohio	All
Number of focus groups	3	3	3	3	12
	n (% of group)	n (% of group)	n (% of group)	n (% of group)	n (% of total)
Total participants	26 (100)	25 (100)	27 (100)	27 (100)	105 (100)
Age					
41–54	25 (96.2)	9 (36.0)	20 (74.1)	13 (48.1)	67 (63.8)
55–67	1 (3.8)	16 (64.0)	7 (25.9)	14 (51.9)	38 (36.2)
Mean (SD)	49.0 (4.0)	56.4 (5.9)	51.6 (5.5)	55 (6.4)	53.0 (6.3)
Race					
White	12 (46.2)	14 (56.0)	16 (59.3)	18 (66.7)	60 (57.1)
African American	12 (46.2)	11 (44.0)	9 (33.3)	9 (33.3)	41 (39.0)
Other	2 (7.7)	0 (0.0)	2 (7.4)	0 (0.0)	4 (3.8)
Education					
High school or less	1 (3.8)	12 (48.0)	9 (33.3)	15 (55.6)	37 (35.2)
Some college or more	25 (96.2)	13 (52.0)	18 (66.7)	12 (44.4)	68 (64.8)
Pack-years smoked					
< 30	6 (23.1)	5 (20.0)	10 (37.0)	2 (7.4)	23 (21.9)
≥ 30	20 (76.9)	20 (80.0)	17 (63.0)	25 (92.6)	82 (78.1)
Mean (SD)	35.2 (10.2)	38.2 (16.2)	35.5 (15.1)	46.4 (19.5)	38.9 (16.4)
NLST-eligible^a^					
Yes	1 (3.8)	15 (60.0)	6 (22.2)	14 (51.9)	36 (34.3)
No	25 (96.2)	10 (40.0)	21 (77.8)	13 (48.1)	69 (65.7)

*SD* Standard deviation

^a^Refers to participants who would meet the eligibility criteria for the National Lung Screening Trial (i.e., age ≥ 55 years and pack-years ≥30)

### Previous cessation attempts and reasons for relapse

All participants were current smokers, but most reported at least one, and often multiple, previous cessation attempts. Many participants tried to quit smoking without cessation aids (i.e., “cold turkey”), while others used an array of methods, ranging from traditional (e.g., nicotine gum/patches, prescription drugs) to less conventional (e.g., hypnosis, acupuncture). Participants’ reasons for relapsing varied, but most were related to a stressful event or time period, such as job loss or relationship problems. One participant explained, “You know that saying ‘when it rains, it pours’? I just had a bad day one day, from the time I got up until that evening, it was just a bad day with my grandchildren, my children, my mom, everybody, and I just couldn’t take it no more and I just ran out the door with 50-cents to buy a cigarette.”

### Perceptions about and experience with lung cancer screening

When asked about lung cancer screening tests, participants had little to no awareness that such tests existed. Data reflecting participants’ limited knowledge about each specific test are provided below.

After the facilitator showed pictures of equipment used during each type of screening test, participants relayed general knowledge or experience with the test. However, the experiences they described were often related to diagnostic procedures, not screening exams. For example, when asked about lung cancer screening with chest x-ray, one participant said, “Yeah… I had a real bad cough and I guess [my doctor] said, ‘Go get a chest x-ray.’” .

#### CT scans

Only two participants in the focus groups were aware that CT scans could be used for lung cancer screening. One learned about it from a diagnostic imaging center representative: “He said if you smoke, even if you have to pay for it out-of-pocket, a CAT scan will show early detection.” Other participants described instances in which lung abnormalities were detected by CT scan, but the detection occurred as a result of a doctor ordering the procedure for an unrelated condition. As one participant explained, “When I had my clot issue [in my heart]… I got a CT scan but they found a spot on the lower left lobe of my lung.” Others knew that a CT scan could be used as a diagnostic procedure for a variety of conditions. For example, one participant said, “I’ve heard of [CT scan] being used when something is wrong and they’re trying, and they’re actually looking [for something].”

#### Chest X-rays

A small number of participants knew about chest x-rays as a lung cancer screening test. Some said their doctor regularly prescribed chest x-rays because of their smoking history: “When I was younger… [doctors] always referred me to get a chest x-ray at least once a year.” Others said they were screened less frequently: “That’s a chest x-ray. That’s the only [lung cancer screening test] I know about. I get one every five years.” Regardless of the frequency of the screening, participants understood that chest x-rays lacked sensitivity as a screening test. One participant suggested that a person had to be “practically gone” with lung cancer for it to be detected by chest x-ray. Another said, “…the doctor told me if you was eat up with cancer, it would show up on [chest x-ray], but he said it’s not a good way to screen for it.” Another participant explained, “The only thing that I’ve heard [about chest x-ray] is that if there is a mass it will show up, but it’s not really a true test. I mean, it’s not a true screening.”

In general, participants who had been screened with chest x-rays recalled limited communication with their doctors about the test and its effectiveness. As one participant remembered, “Well, basically… [chest x-ray] really doesn’t show a whole lot. It would show maybe some abnormalities. [The doctors] were very vague about it.” Participants also recalled only limited communication about their results. One participant said, “I get a letter two, three weeks later…with the results. I may not necessarily know what they mean but I figure if [the doctor’s] not calling me, then everything’s all right.”

#### Sputum cytology, Spirometry, and pulmonary function tests

No participant had heard of sputum cytology as a potential lung cancer screening test. However, some suggested that doctors could screen for lung cancer by spirometry (i.e., the doctor holds a stethoscope to the patient’s chest and listens to him/her breathe) or by pulmonary function tests (i.e., the patient blows into a machine that measures the volume and speed of air flow).

### Desire to pursue lung cancer screening

Most participants learned about lung cancer screening for the first time during the focus groups. As the discussions concluded, many participants expressed interest in pursuing lung cancer screening. One participant said, “I think I’m going to try to find out more about [lung cancer] screening because I do smoke and I’m constantly scared.” Other participants suggested that doctors should redirect their patient education efforts from smoking cessation to lung cancer screening: “At this point in my life, I want my doctor to say, ‘You know what, you’ve been smoking these cigarettes. I want to start doing this testing on you to make sure that cancer’s not lurking somewhere.’” .

Some participants who had not previously heard about lung cancer screening expressed frustration that their doctors had not told them about it. One participant said,

“I’m thinking I’m going to ask my doctor why hasn’t she not only offered [lung cancer screening] to me, but at least given me the option, tell me what [lung cancer screening tests] are and what they consist of and what the benefits would be and let me make the decision. Or even just to see if my insurance would cover it. Because it’s something that I would love to have done.”

Another participant suggested that doctors may purposely withhold information about lung cancer screening: “Maybe they just figure you deserve [lung cancer] since you smoke.” As previously noted, participants were told at the end of the focus groups that the USPSTF did not recommend lung cancer screening at that time.

### Perceived barriers to lung cancer screening

Although many participants expressed an interest in lung cancer screening, others had concerns that could be potential barriers to screening. We identified seven barriers to screening at three levels of the social ecological model (Table 2): cost of procedure (CT specifically) and confusion around screening results at the health care system level; fatalistic beliefs and distrust of the medical system at the cultural level; and lack of knowledge about screening, denial of risk for lung cancer, and concerns about the screening procedure at the individual level. Table 2 provides illustrative quotes for each barrier.

**Table 2:** Perceived barriers to lung cancer screening and illustrative participant quotes, by social ecological model level

Health care system level barriers	
Cost of procedure	“I actually scheduled [a screening] and then they called me back and told me I needed to bring $300 with me. And I just didn’t have it for something that was not wrong with me.”
	“Insurance, you know. More than likely unless there is something that calls for [a CT scan], to just to go in and say you’d like to have one, on the safety side, insurance won’t pick it up.”
	“The cost, yeah. [Doctors] don’t just say, ‘Oh, let’s go have a CAT scan to see if you got cancer.’”
	“It probably comes from the insurance companies. Why should the doctor put you out that way because you may end up having to pay for the test in the first place because you smoke. Insurance companies don’t want to pay for anything.”
Confusion around test results	“A lot of times [screening tests] don’t really see things because like I just had a mammogram. Then they said, ‘We want you to go over here and get an ultrasound. Then we want you to go over here,’ you know.”
	“…you have just put me through all this mind bending and tests and running all over the place. I’ve been a stress mess. Tell me once and for all, do I have something or do I not? And I don’t believe that those tests can do that.”
Cultural level barriers	
Fatalistic beliefs	“It’s part of the fear of having it done and then finding out that you do have it [lung cancer]. I don’t know if that’s true but everybody that I’ve come across, if they find out they have it then you’re going to have it in your mind, ‘Oh, my God, I have it. I’m going to die.’”
	“She said they thought they found, saw something…they wanted to run more tests. I just didn’t show back up because I feel like if it’s my time, I don’t want to know about it.”
	“[The CT]‘s the one I would feel comfortable with, but me, in general, I’m like what I don’t know won’t hurt me.”
Distrust in medical system	“I’m kind of like the person that thinks doctors and insurance companies are in cahoots anyway. And every time you go to the doctor, it doesn’t matter what you go for, they want you to have this test. They want you to have that test. They want you to be checked for this and that and I’ve done it for so many years. That’s why I don’t do it anymore unless it’s just absolutely necessary.”
	“I don’t trust [my doctor]…Mine’s the in and out, I’ll push you in, push you out, push you in, push you out…. He don’t got time for me. He don’t touch me….He won’t look at me. He prints out the [prescriptions] on a laptop. They’re shot straight to CVS. I’m in and out.”
	“It’s like if you’re not hurt in that area, it’s avoided…. You’re checking the blood and doing this and that, but if you’re not hurting in that particular area of the body, they’re not going to ask you. It’s like they want to spend less money on you as possible.”
Individual level barriers	
Lack of knowledge	“...why haven’t we heard [about] it? I mean, in this age of the Internet and news, why is that something that’s not [known]. There’s plenty of medical shows on [television].”
Denial of risk	“I’m concerned about [lung cancer] but I don’t think about it…who knows if cigarettes actually cause that [cancer]? I mean, there’s a lot of people that have cancer that never smoked.”
	“I’m just getting ready to turn 50 and I keep thinking that’s not going to happen ‘til I’m over 70 or 80…So you just kind of, I guess, denial.”
	“I don’t want to know nothing else. Don’t hear no rattle. Don’t hear no bad news. I’m fine.”
Fear of screening procedure	“…when I had [a CT] they had to give me something to calm me because that little tube was too much enclosure….And I got claustrophobia.”
	“…an X-ray is… so much more relaxed. I don’t like MRIs and CAT scans…I’d rather stand in front of an X-ray machine any day.”

## Discussion

This qualitative study found that smokers had limited knowledge of lung cancer screening. Moreover, study participants who were aware of lung cancer screening most often described experiences with chest x-ray, a test that has been shown to be ineffective for lung cancer screening [12]. Only two of the 105 participants in our study were familiar with the use of a CT scan for lung cancer screening. The general lack of awareness of CT scan as a screening test for lung cancer is consistent with more recent qualitative research, indicating that lack of knowledge may continue to be an issue for screening uptake. [13, 14]. As screening with low-dose CT becomes more available, research should continue to assess whether eligible smokers are aware of the availability of screening. Although several states had few or no lung cancer screening centers shortly after the recommendations were released [15], the national landscape for lung cancer screening is likely to change in the coming years [16].

Although most participants were unaware of lung cancer screening when the focus groups began, many expressed an interest in pursuing it as an option. Nearly all had tried to stop smoking at least once using a variety of approaches, leading some to even suggest that cessation efforts be redirected toward screening. These sentiments reflect the need for doctors to communicate the limitations of low-dose CT scans and the process and risks associated with follow-up diagnostic testing. However, the participants who had experience with lung cancer screening tests recalled only limited communication with their doctors about the procedure, echoing findings of another study [17]. The 2015 CMS coverage decision attempts to address this issue by requiring an initial visit for counseling and shared decision making. Specifically, it states that this visit should include the following: "1) determination of beneficiary eligibility; 2) the use of one or more decision aids, to include benefits and harms of screening, follow-up diagnostic testing, over-diagnosis, false positive rate, and total radiation exposure; 3) counseling on the importance of adherence to annual lung cancer low-dose CT screening, impact of comorbidities and ability or willingness to undergo diagnosis and treatment; and 4) counseling on the importance of maintaining cigarette smoking abstinence if former smoker; or the importance of smoking cessation if current smoker and, if appropriate, furnishing of information about tobacco cessation interventions…" [4]. The website of the American College of Radiology includes sample forms and other resources for meeting and documenting these CMS requirements [18]. A recent study found that decision aids were indeed effective in increasing low income patients’ understanding of the potential for false positives and extra testing associated with lung cancer screening [19].

About two-thirds of our study participants were smokers who would not meet the 2013 USPSTF or 2015 CMS eligibility criteria for screening. Their interest in lung cancer screening suggests that doctors may need to be prepared to respond to questions from both those who meet the eligibility criteria for screening and those who do not.

Despite the difficulties with quitting smoking that our participants reported, smoking cessation remains the most effective way to prevent lung cancer [20]. To ensure that cessation efforts are maintained as lung cancer screening programs are implemented, researchers need to understand how lung cancer screening test results affects smokers’ cessation attempts. According to a 2010 estimate, approximately 8.7 million current and former smokers in the United States would meet the NLST criteria for lung cancer screening with low-dose CT [21]. Using data from the NLST, one study reported that a false positive screening result was associated with increased smoking cessation and less relapse among recent quitters. Negative screening results, in contrast, were not associated with greater relapse among long-term former smokers [22].

Understanding smokers’ barriers to lung cancer screening with low-dose CT can help to guide intervention efforts and increase appropriate screening. This study used the social ecological model to identify several potential barriers, which are described using participant quotes in Table 2. Recent studies have similarly identified cost and distrust as barriers, but have also found smoking stigma and inconvenience (e.g., scheduling and transportation) to be issues [14]. Further research is needed to determine if these barriers can be generalized to the current US population eligible for screening. For example, increased coverage of payment for lung cancer screening through the 2015 CMS ruling may decrease the relevance of cost as a barrier.

Our study has at least four limitations. First, our findings are limited to participants from two US cities in 2009; smokers in other communities may have different perceptions, and smokers’ perceptions may have changed following the 2013 USPSTF and 2015 CMS recommendations. Nevertheless, this study may serve as a baseline for changes in smokers’ knowledge and experience. Second, our criteria to select focus group participants were different from the 2013 USPSTF and 2015 CMS eligibility criteria for lung cancer screening. For example, nearly one fifth of our participants had a < 30 pack-year smoking history. It is possible that these smokers with a shorter smoking history would be less aware of lung cancer screening than those who meet the 30 pack-year requirement. Third, although our study included a larger proportion of African Americans (39.0%) than the NLST study (4.5%), our study design did not allow examination of results by race or ethnicity [23]. Previous research suggests, however, that African Americans are more likely than Whites to avoid screening due to fear of the disease [24]. This idea supports the barrier related to fatalistic beliefs that was identified in this study. Fourth, nearly two thirds of our study had at least a college education, indicating that this group was particularly highly educated. Nevertheless, the lack of knowledge about lung cancer screening and the desire to learn more has been similarly noted in qualitative studies of low income, less educated patients [19].

## Conclusion

Although the smokers in this study had little experience with and knowledge about lung cancer screening, their interest in exploring it as an option was clear. Implementation of lung cancer screening should include sufficient education and tools to improve patient understanding of the benefits and risks of screening. Additional qualitative studies that examine smokers’ knowledge about lung cancer screening are also recommended as screening becomes more widely used in the United States. At the same time, researchers and health care providers must continue to make smoking cessation a priority because it is the most effective way to prevent lung cancer.

## Additional file

Additional file 1: Discussion Moderators’ Guide for Smokers. Description of Data: Semi-structured moderator guide used to conduct focus groups of smokers for this study. File Format: Adobe PDF (.pdf). (PDF 224 kb)

## Abbreviations

CDC: Centers for Disease Control and Prevention
CMS: Centers for Medicare & Medicaid Services
CT: Computed tomography
NLST: National Lung Screening Trial
RTI: Research Triangle Institute
USPSTF: United State Preventive Services Task Force
