# Supplementary material for: A qualitative analysis of smokers’ perceptions about lung cancer screening
Source: BMC Public Health. 2017 Jun 21;17:589. doi: 10.1186/s12889-017-4496-0 (PMC5479014; doi:10.1186/s12889-017-4496-0)
Supplement: Additional file 1: — Discussion Moderators’ Guide for Smokers. Description of Data: Semi-structured moderator guide used to conduct focus groups of smokers for this study. File Format: Adobe PDF (.pdf). (PDF 224 kb) [file 12889_2017_4496_MOESM1_ESM.pdf]

Form Approved
OMB No. 0920-0801
Expiration Date: 01/31/2011

APPENDIX I-1: DISCUSSION MODERATORS GUIDE FOR SMOKERS

MODERATOR'S GUIDE FOR HEALTH CARE CONSUMER FOCUS GROUPS

Consumers' Practices regarding Preventive Health Behaviors and Screenings
Moderator Discussion Guide

Revised 6/10/09

I. BACKGROUND AND INTRODUCTIONS (@ 20 minutes)

A. Background (@10 minutes)

Hello. My name is ____ and I work with ____, a consulting firm. I want to thank you for participating today. We are working with the U.S. Centers for Disease Control and Prevention, or CDC, to understand what people do to take care of their health and learn about what their doctors tell them and recommend with regard to preventive health care and health screening tests. My role is to guide our discussion today and to encourage everyone to share their thoughts and ideas.

The group discussion will take about two hours. Your participation in this discussion is voluntary and you can end your participation and leave the group at any time. The discussion will be audio recorded for note taking purposes. Observers, including someone from CDC, are also present behind the mirror so that they hear your opinions directly. Your name will not be connected to any of the written notes or transcripts. Everything you say will be kept private. Results will be presented without any names and quotes will not be attributed to any specific individual.

We have some general rules for our discussion today. We ask that you only use first names when addressing yourself and others. Also, as mentioned on the consent form you signed, we ask that you respect each others' privacy and do not share what is said in the group once it is over. Please set any phone or other ringing devices to vibrate. If you need to take a call, please step out to do so and join us once you are done. [MODERATOR GIVES DIRECTIONS TO THE RESTROOMS].

Do you have any questions for me before I ask you to introduce yourselves?

Public reporting burden of this collection of information is estimated to average 2 hours per response, including the time for reviewing instructions, searching existing data sources, gathering and maintaining the data needed, and completing and reviewing the collection of information. An agency may not conduct or sponsor, and a person is not required to respond to a collection of information unless it displays a currently valid OMB control number. Send comments regarding this burden estimate or any other aspect of this collection of information, including suggestions for reducing this burden to CDC/ATSDR Reports Clearance Officer; 1600 Clifton Road NE, MS D-74, Atlanta, Georgia 30333; ATTN: PRA (0920-0801)

B. Participant Introductions (@ 10 minutes)

I'd like each of you to introduce yourself, and have you name one thing you do to take care of your health that is a "good" habit and one thing you do, or don't do, that is a "not so good" habit. Let's hear what you have to say. I'm going to record your answers. Just to reiterate, your names will be removed from all information collected.

Probe, if necessary: For instance, maybe you try to eat right, but you don't exercise as much as you'd like, or you rarely go to the doctor.

[MODERATOR RECORDS RESPONSES ON AN EASEL USING TWO SHEETS – ONE FOR THE GOOD THINGS AND ONE FOR THE BAD ONES. AT THE END OF THE EXERCISE, MODERATOR VERBALLY SUMMARIZES RESPONSES.]

II. HEALTH CONCERNS AND PREVENTIVE HEALTH BEHAVIORS (@ 35 MINUTES)

You already know that taking care of your health is our main topic. I'd like to begin by asking you two questions and having you write down your answers. [MODERATOR PASSES OUT PAPER AND PENS]

The reason I am asking you to write down your answers is so that we get your first reaction to the question. Then we will spend some time talking about your answers and the answers from the rest of the group. Please keep in mind that sometimes in group discussions, people can be reminded about something when they hear someone else talking about it. That's ok if that happens, but we'd really like to hear your *first* reaction because we'll be using your answers to help guide today's discussion.

Questions:

1. What's the health problem you are most worried about getting in the future?

2. What health screening tests do you get on a regular basis? If you are unsure what we mean here, just put down your best guess.

[NOTE TO MODERATOR – KEEP THE DISCUSSION FOR THIS INTRO ACTIVITY BRIEF.]

<u>Future Health Concerns (@ 15 minutes)</u>

3. Let's go over our first question. What were some of the health concerns you have for the future? Anyone want to volunteer?

Probe: Has anyone else listed the same concern?
Probe: Any other concerns?

[MODERATOR RECORDS RESPONSES ON EASEL AND AT THE END OF THE EXERCISE SUMMARIZES RESPONSES]

Preventive Health Behaviors (@ 20 minutes)

4. Let's move on to the next question. What are some of the screening tests you have gotten on a regular basis? I'm going to make a list and then we'll come back and discuss some of these in depth. [MODERATOR RECORDS RESPONSES ON EASEL].

Probe: Did anyone else list the same test?
Probe: Other tests?
Probe: What about…

- blood pressure
- cholesterol levels/ tests for heart disease
- colorectal cancer screening tests
  - Probe: which tests? Take-home stool tests? Colonoscopy?
- blood sugar testing for diabetes
- EKG or other heart tests
- [MALE GROUPS] PSA for prostate cancer
- [FEMALE GROUPS] mammograms for breast cancer
- [FEMALE GROUPS] pap smear for cervical cancer

[NOTE TO MODERATOR: DO NOT PROBE ON THESE TESTS REGARDLESS OF IF THEY COME UP OR NOT]

- Dental exams
- Vision
- Hearing
- Skin exams
- Sputum test

[MODERATOR VERBALLY SUMMARIZES FOR THE GROUP CONNECTIONS BETWEEN HEALTH CONCERNS, HABIT AND TESTS MENTIONED].

III. DIET-RELATED HEALTH ISSUE (@ 5 minutes)

Several of you mentioned you are concerned about [INSERT DIET-RELATED HEALTH CONCERN FROM QUESTION 1. NOTE TO MODERATOR, IF POSSIBLE USE A SPECIFIC CONCERN SUCH AS EATING TOO MUCH SUGAR OR FAT.]

5. What, if anything, has your doctor told you about [DIET-RELATED HEALTH CONCERN]?

Probe: Which tests listed here [MODERATOR REFERS TO LIST OF TESTS FROM PREVENTIVE HEALTH QUESTION – EXAMPLES CHOLESTEROL TEST OR BLOOD SUGAR TEST] are related to [DIET-RELATED HEALTH CONCERN – CHOLESTEROL OR DIABETES]?

6. What has your doctor told you about tests related to [DIET-RELATED HEALTH CONCERN – CHOLESTEROL OR DIABETES]?

    [NOTE TO MODERATOR – WANT TO KEEP THIS SECTION UNDER 5 MINUTES. IF TIME PERMITS ASK PROBES BELOW]
    Probe: What did they tell you about the tests?
    Probe: Was that test done as part of your routine care / check-up?
    Probe: Or did you have that test because of a particular problem / symptom?

[NOTE TO MODERATOR: IF THE MAJORITY OF THE PARTICIPANTS ARE UNDER AGE 50, DO NOT ASK Q7 AND 8 AND SKIP TO SECTION IV SMOKING]

7. What, if anything, has your doctor or other health care provider told you about colon cancer and colon cancer screening?

8. What has your doctor or other health care provider told you about screening tests for colorectal cancer?
    Probe: Was a screening test recommended to you?
    Probe: What screening test was recommended?
    Probe: Was the test done as part of your routine care or check-up?
    Probe: Or did you have that test because of a particular problem / symptom?

IV. SMOKING (@ 45 minutes)

[IF SMOKING WAS NOT MENTIONED AS A BAD HABIT IN INTRODUCTIONS, THEN ASK 9A]
[IF SMOKING WAS LISTED AS A BAD HABIT INTRODUCTIONS, THEN ASK 9B]

9A. When we introduced ourselves and discussed our health habits, I noticed no one mentioned smoking. Does anyone have any thoughts about that?

    Probe: Does your *doctor* or other health care provider know that you smoke? (If yes), What does your doctor or other health care provider tell you about your smoking?
    Probe: What has your *doctor* or other health care provider told you about health tests for smokers?

    GO TO Q. 10.

9B. When we introduced ourselves and discussed our health habits, several of you/one of you mentioned that one of your "not so good" habits was smoking. Does your *doctor*

or other health care provider know that your smoke? (If yes) - What does your doctor or other health care provider tell you about your smoking? DO ROUND ROBIN OF THESE RESPONSES.

Part of the reason you are here is because you've all been to the doctor in the past two years. Tell me about last doctor's visit.

PROBE – Any strategies / resources doctor specifically mention? \
PROBE – What types of things does your doctor say about your smoking?
PROBE – Aware of resources in area – Quit Now North Carolina which is a quitline, Carolina Medical program, Presybestrian Hospital.

We know there are screening tests for some types of cancer such as breast and prostate cancer, which screening tests for lung cancer are you aware of, if any?

10. Did your doctor ever talk to you about screening tests for your lungs – that is, screening tests for lung cancer?

IF SOME PARTICIPANTS SAY YES, ASK

10a. What did the doctor or health care provider say?
10b. Was there a particular test they brought up? [IF YES, THEN GO TO THE QUESTIONS FOR THE SPECIFIC TEST UNDER 10d1, 2, OR 3.]

IF ALL PARTICIPANTS SAY NO, ASK

10c. Just to confirm, no one has had any screening tests to check your lungs?

Let me just double check with the names of a few tests… *[NOTE TO MODERATOR ASK ALL THREE, IN THIS ORDER]*

10d1. Chest x-ray? [SHOW PICTURES]

[IF YES ASK]

- What did the doctor or other health provider tell you about the test?
- Did you ask for the test or did they recommend the test to you?
  [IF RECOMMENDED]
  - What did your doctor tell you about why you needed the test?

  [IF TEST PERFORMED]
  - Was that test done as part of your routine care / check-up?
    Probe: Or did you have that test because of a particular problem / symptom?
    Probe: What symptoms or problems were you experiencing?
  - What happened after the test?

- Did you have any follow-up tests?
  Probe: What tests?
  Probe: How often?
- Anyone else ever had a chest x-ray for lung cancer screening?
  Probe: How was your experience similar or different?

[ALL PARTICIPANTS]

- Other than from your doctor or health care provider, what have you heard about chest x-ray as a screening test for lung problems?
  Probe: Where did you hear this?
  Probe: Media, American Cancer Society, National Cancer Institute
  Probe: What do you think about what you heard?

[NOTE TO MODERATOR - ASK ABOUT OTHER TWO TESTS IN Q10]

10d2. CAT or CT scan? <u>[SHOW PICTURES]</u>

Probe: Are you familiar with what a CAT scan is? [REGARDLESS OF RESPONSE, READ DEFINITION -A CAT scan is a series of detailed pictures of areas inside the body taken from different angles. The pictures are created by a computer linked to an x-ray machine. Also called CT scan, computed tomography scan, computerized axial tomography scan, and computerized tomography.]

[IF YES ASK]

- What did the doctor or other health provider tell you about the test?
- Did you ask for the test or did they recommend the test to you?

[IF RECOMMENDED]

- What did your doctor tell you about why you needed the test?

[IF TEST PERFORMED]

- Was that test done as part of your routine care / check-up?
  Probe: Or did you have that test because of a particular problem / symptom?
  Probe: What symptoms or problems were you experiencing?
- What happened after the test?
- Did you have any follow-up tests?
  Probe: What tests?
  Probe: How often?
- Anyone else ever had a CAT scan?
  Probe: How was your experience similar or different?

[ALL PARTICIPANTS]

- Other than from your doctor or health care provider, what have you heard about CAT scan as a screening test for lung problems?
  Probe: Where did you hear this?
  Probe: Media, American Cancer Society, National Cancer Institute
  Probe: What do you think about what you heard?

[NOTE TO MODERATOR - ASK ABOUT OTHER TWO TESTS IN Q10]

10d3. Sputum sample/cytology? [SHOW PICTURES]

IF NEEDED, PROVIDE THE FOLLOWING DEFINITION OF A SPUTUM SAMPLE: A SPUTUM SAMPLE REFERS TO THE COLLECTION OF THE MUCUS THAT IS COUGHED UP FROM THE LOWER AIRWAYS.

[IF YES ASK]

- What did the doctor or other health provider tell you about the test?
- Did you ask for the test or did they recommend the test to you?
  [IF RECOMMENDED]
  - What did your doctor tell you about why you needed the test?

  [IF TEST PERFORMED]
  - Was that test done as part of your routine care / check-up?
    Probe: Or did you have that test because of a particular problem / symptom?
    Probe: What symptoms or problems were you experiencing?
  - What happened after the test?
  - Did you have any follow-up tests?
    Probe: What tests?
    Probe: How often?
  - Anyone else ever had a sputum sample/cytology test?

  Probe: How was your experience similar or different?

[NOTE TO MODERATOR – IF NOT YET COVERED, ASK ABOUT OTHER TWO TESTS IN Q10]

11. Has anyone had any other tests to look for lung problems?

    [IF YES ASK]
    - What did the doctor or other health provider tell you about the test?

- Did you ask for the test or did they recommend the test to you?
- [IF RECOMMENDED] What did your doctor tell you about why you needed the test?
- Was that test done as part of your routine care / check-up?
  Probe: Or did you have that test because of a particular problem / symptom?
  Probe: What symptoms or problems were you experiencing?
- What happened after the test?
- Did you have any follow-up tests?
  Probe: What tests?
  Probe: How often? Anyone else ever had [INSERT TEST]?
  Probe: How was your experience similar or different?

NEW QUESTION:
People who smoke often say that they've quit smoking at different periods during their lifetime and then go back to smoking. Can you tell me about a time when you've quit smoking even if it was for a short period of time.

PROBES:

How long did you quit?
What caused you to want to quit?
What did you use to do it?
What was successful?
Did someone help you quit?
What triggered your relapse? PROBE: Stress

Sometimes people are more likely to smoke or continue to smoke if their spouse or significant others or close friends smoke. Tell me about individuals in your life who play a role in your smoking.

III. SUMMARY AND WRAP UP (@ 5 min.)

We are just about out of time now. I'd like to close with two questions for which I would like you to write down your answer while I check on your paperwork.

Question 1: If you could give one piece of advice to people who provide health care to people who smoke, what would it be? "For example, I would like it if my doctor did or did not..."

Question 2:
If you were to design an ad to help people quit smoking, what would if look like? PROBE: Images, key words, messages.

MODERATOR DISCUSS ANSWERS
Thank you for being a part of the group. Your feedback is very important to CDC. I'll show you out and direct you to the person who will provide you with your

payment for your time today. Here is some information to take with you today. [MODERATOR HANDS OUT POSTCARD].

[MODERATOR INQUIRES ABOUT ANY “TAKE AWAY” MESSAGES PARTICIPANTS HAVE FROM BEING IN THE GROUP]

Please note - While lung screening with any of the tests we discussed today is not currently recommended by the U.S. Preventive Services Task Force (USPSTF), studies are currently underway to assess the effectiveness of CAT.

TOTAL TIME: @ 120 minutes
